# Reinforced Concrete Slabs Strengthened with Carbon Textile Grid and Cementitious Grout

**DOI:** 10.3390/ma14175046

**Published:** 2021-09-03

**Authors:** Hyeong-Yeol Kim, Young-Jun You, Gum-Sung Ryu

**Affiliations:** Structural Engineering Department, Korea Institute of Civil Engineering and Building Technology (KICT), Goyang-Si 10223, Korea; hykim1@kict.re.kr (H.-Y.K.); ryu0505@kict.re.kr (G.-S.R.)

**Keywords:** carbon textile, cementitious grout, concrete structure, flexural strengthening, textile reinforced concrete (TRC), fabric reinforced cementitious matrix (FRCM), structural testing

## Abstract

A textile reinforced concrete (TRC) system has been widely used for repair and strengthening of deteriorated reinforced concrete (RC) structures. This paper proposes an accelerated on-site installation method of a TRC system by grouting to strengthen deteriorated RC structures. Four RC slabs were strengthened with one ply of carbon textile grid and 20 mm-thick cementitious grout. The TRC strengthened slab specimens were tested under flexure and the test results were compared with those of an unstrengthened specimen and theoretical solutions. Furthermore, the TRC strengthened specimens experienced longer plastic deformation after steel yield than the unstrengthened specimen. The TRC strengthened specimens exhibited many fine cracks and finally failed by rupture of the textile. Therefore, TRC system with the proposed installation method can effectively be used for strengthening of deteriorated RC structural elements. The theoretically computed steel yield and ultimate loads overestimate the test data by 11% and 5%, respectively.

## 1. Introduction

Textile grid reinforcement has been widely used for repair and strengthening of deteriorated reinforced concrete (RC) structures [1,2,3]. The textile grid is generally integrated with a cementitious binder to form a composite material. If the cementitious binder contains coarse aggregates, we refer to it as Textile Reinforced Concrete (TRC). On the other hand, if cementitious mortar with no coarse aggregates is used, we refer to it as Textile Reinforced Mortar (TRM). TRM and TRC are also referred to as Fabric Reinforced Cementitious Matrix (FRCM) in the literature [4]. 

In the past two decades, extensive investigations, mainly experimental studies, have been conducted on the strengthening of RC structures with TRC systems. Previous studies examined the flexural strengthening [5,6,7,8,9] and shear strengthening [10] of structurally deficient RC beams by TRC systems. In these studies, polymeric coating and sand coating of textile [5], U shaped textile anchors installed at the edges of the beam [6,10], the pre-tensioning of textile [7], the matrix composition and strength [8], and the number of textile plies [9,10] were considered as design variables. These studies verified that when the number of textile plies was increased, the structural performance improved, but the improvement was rarely proportional to the number of plies. The introduction of pre-tensioning of textile and textile anchors enhanced the strengthening effect. On the other hand, the strength of the matrix showed an insignificant influence on the strengthening effect.

Flexural strengthening of RC slab-type elements with the TRC system has also been investigated. Schladitz et al. [11] tested 6.75 m-long RC slabs strengthened by a carbon TRC system and their test results showed a fair correlation with the analytical calculation. Loreto et al. [12] demonstrated that the behavior of flexural strengthened RC slab by a TRC system can be estimated by analytical calculation, if the tensile properties of the TRC system can be accurately obtained from coupon tests. An RC bridge deck slab segment [13] and a two-way RC slab [14] have been strengthened by carbon TRC systems considering the number of textile plies as well as the matrix composition and strength as design variables and tested. These studies verified that the carbon TRC system is very effective for flexural strengthening of RC slabs. More recently, a research group in KICT conducted a series of investigations on strengthening for RC slabs by carbon TRC systems. In their studies, RC slabs were strengthened with cast-in-place carbon TRC system [15,16]. Meanwhile, RC slabs were also strengthened with a precast TRC panel with grout [17]. 

The TRC system has also been used for strengthening of infill walls (masonry walls) [18,19] for increasing the confinement of RC columns [20,21]. The influence of matrix type, fiber type and orientation, fiber surface treatment, and number of textile plies on the structural performance of RC structural elements strengthened with TRC systems is well summarized in the literature [1,2,3,15]. 

The results of previous investigations confirm that a properly designed and installed TRC system can effectively be used for repair and strengthening of deteriorated RC members. However, a lack of design guidelines for TRC systems was one of the technical barriers to conduct practical applications. Nonetheless, design guidelines and testing methods for TRC systems in repair and strengthening of RC structures have gradually been established in recent years [4,22,23,24]. 

**Figure 1:**
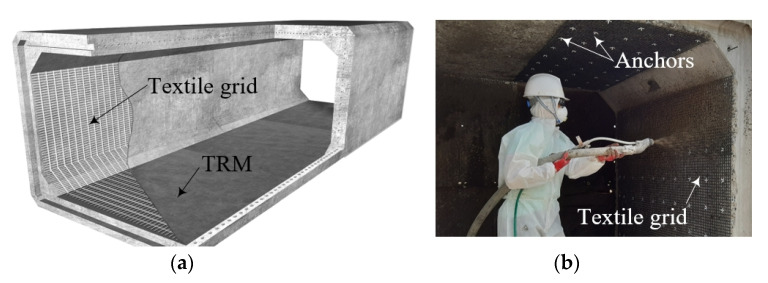
Typical installation methods: (**a**) hand lay-up; and (**b**) shotcreting (spraying).

Typical on-site installation method of a TRC system for the strengthening of RC structures is a hand lay-up method (Figure 1a). A shotcreting or spraying method (Figure 1b) is preferred for a TRC strengthening site that requires a fast installation or involves a large area of application [25]. A research group in KICT developed an accelerated installation method for RC structures with difficult accessibility or with a narrow working space using precast TRC elements with cementitious grout [17,26,27,28,29]. In the developed method, the precast TRC panel element is externally bonded to an existing RC structure by grouting. However, the main shortcoming of the developed method over conventional on-site cast-in-place (CIP) TRC installation methods is that the size of the precast TRC panel is limited and hence an effective on-site connecting method for the precast elements should developed. In a preceding study [17], an on-site connecting method for precast TRC panels with a lap-spliced textile grid and grout was proposed. 

**Figure 2:**
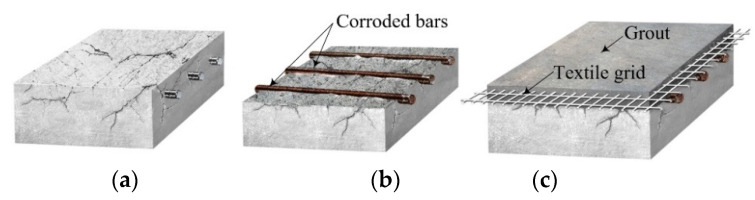
(**a**) deteriorated concrete element; (**b**) removing deteriorated concrete cover; and (**c**) installation of TRC system with grout.

In this paper, an on-site installation method for a TRC system with grouting is proposed for strengthening of deteriorated RC structures shown in Figure 2a. The present study assumes that corrosion of reinforcing steel is the main cause of deterioration in RC structures and deteriorated RC structures can be repaired or strengthened by a TRC system.

The proposed method of TRC installation can be completed by the following four steps: (1) removing the deteriorated concrete cover (Figure 2b); (2) installation of textile grid by anchors; (3) building of formwork; and (4) grouting (Figure 2c). 

In a preceding study [17], precast TRC panels were used as a formwork as well as reinforcement. On the other hand, the proposed installation method of TRC system uses a plywood formwork. The main advantage of the proposed method over the precast TRC panel, an on-site connection of TRC system can easily be obtained with a lap-spliced textile grid and grout. 

The objectives of this paper are to strengthen RC slabs with a TRC system by the proposed installation method and to experimentally validate the structural performance of the strengthened slab specimens by a failure test. To validate the proposed installation method of TRC system, five 2000.0 mm-long and 500.0 mm-wide RC slabs were fabricated first; and four of the slabs were strengthened with one ply of carbon grid textile and grout. The RC slabs strengthened by the TRC system with grout were tested by a three-point bending test. The structural performance of the strengthened RC slab specimens is compared with that of the unstrengthened RC slab specimen and the results of analytical calculations. Furthermore, the influence of the strengthening method on load carrying capacity and the ductility of the RC slabs strengthened with the TRC system are examined by comparing the test results with those of previous studies. 

## 2. Experimental Program

### 2.1. Fabrication of RC Slabs

**Figure 3:**
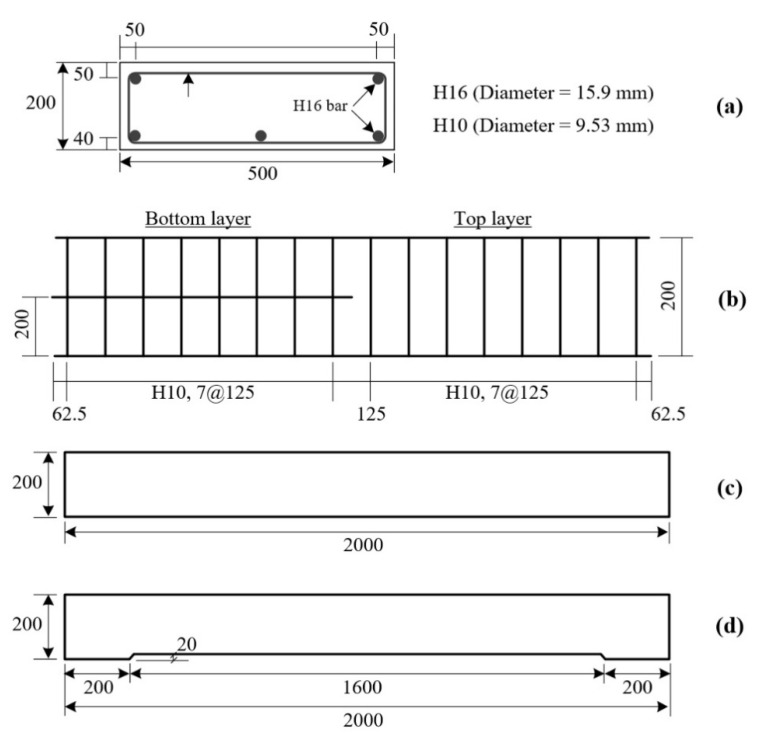
RC slabs: (**a**) cross-sectional dimensions; (**b**) reinforcement details; (**c**) side view of RC slab; and (**d**) side view of RC slab with indented space (unit: mm).

Five 500 × 200 × 2000 mm^3^ (width × height × length) RC slabs were fabricated in this study. Figure 3a,b illustrates the cross-sectional dimensions and arrangements of longitudinal and transverse reinforcing bars, respectively. The yield strength of the 16 mm- and 10 mm-steel bars was respectively 451 MPa and 488 MPa. Figure 3c,d shows the side views of the RC slabs with and without indented space, respectively. The indented space (Figure 3d) underneath the bottom bars of the slab is designed to fill the grouting material. Note that the indented space (depth = 20 mm) is assumed as the deteriorated concrete cover that must be removed prior to TRC strengthening. 

The slabs were casted with a ready-mixed concrete with a design strength of 27 MPa. Table 1 summarizes the mix composition of the concrete used. The slab was cured with a plastic covering in a temperature-controlled room at about 20 °C for 30 days.

### 2.2. Test Specimens

Table 2 lists characteristics of the test specimens fabricated for a flexure test. In the table, the RC specimen is an unstrengthened slab (control) and SG series specimens are strengthened slabs with one ply of carbon textile grid and cementitious grout. 

In this study, as provided in Table 3, a warp-knitted carbon textile grid (Sand coated Q85/85-CCE-21-E4, Solidian-Kelteks Co. Ltd., Karlovac, Croatia) was used as textile reinforcement. A commercially available cementitious non-shrink grout (Chemius Korea Ltd. Co., Seoul, Korea) with a design strength of 50 MPa was used as a binder for the TRC system. The mix composition of the grout is summarized in Table 4. Polypropylene short fibers (0.3%, length = 6.0 mm) were mixed with the grout to mitigate shrinkage-induced crack formation. 

**Figure 4:**
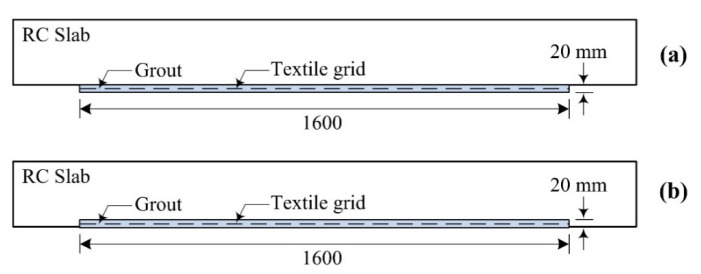
TRC strengthening plan for RC slabs: (**a**) SG-1 series specimens; and (**b**) SG-2 series specimens.

Figure 4 illustrates strengthening plans for the SG series specimens. As shown in Figure 4a, the region underneath the RC slab was directly strengthened by the TRC system with grout and these specimens were labeled as the SG-1 series. On the other hand, for the SG-2 series specimens, the TRC system was cast within the indented space of the RC slab (Figure 4b).

**Figure 5:**
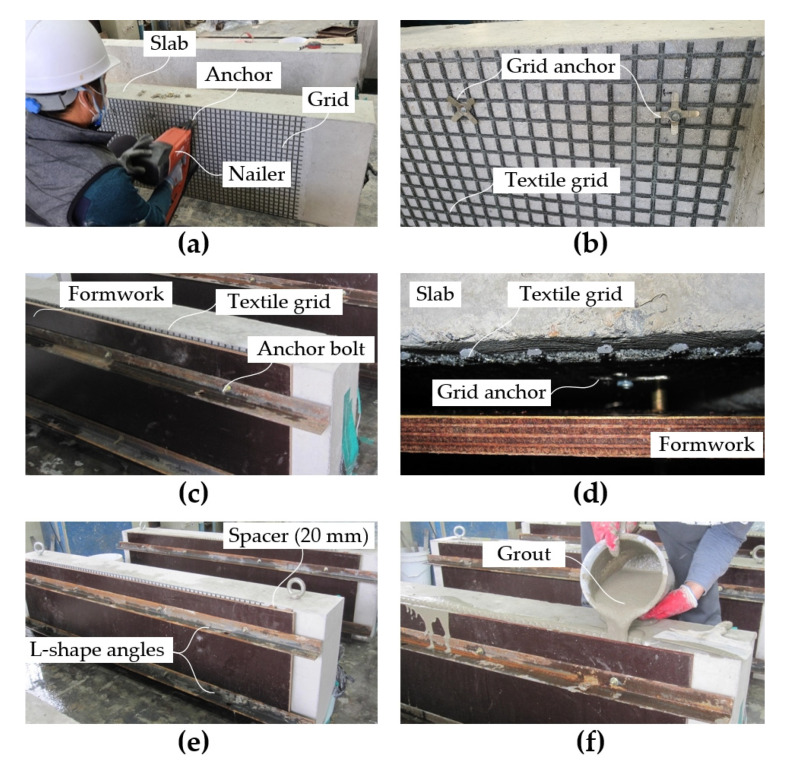
TRC strengthening process: (**a**) installation of textile grid; (**b**) textile grid and anchors; (**c**) formwork for S-1 series specimens; (**d**) grouting space; (**e**) formwork for S-2 series specimens; and (**f**) grout filling process.

**Figure 6:**
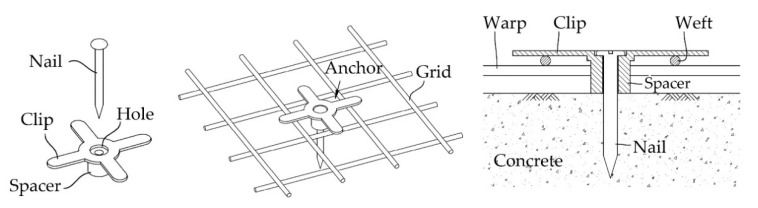
Schematics of a grid anchor system [31,32,33].

Figure 5 depicts the TRC strengthening process for the RC slabs during the fabrication. The RC slab was set vertically onto a steel bed and a 1600.0-long and 500.0 mm-wide textile grid was placed at the bottom face of the slab (Figure 5a). Textile grid anchors were spaced at 200 mm in the horizontal and vertical directions to fix the grid onto the RC slab (Figure 5b). As illustrated in Figure 6, the grid anchor [31,32,33] consists of a cross-shaped clip, a spacer, and a nail. The effectiveness of the grid anchor system has been previously demonstrated by a trial project for strengthening of an RC box structure [16]. The spacer of the anchor system is designed to maintain a minimum space (about 2–3 mm) between textile and concrete during grout filling. Finally, the grid anchor was fixed onto the RC slab with a shooting nail by a gas-powered nailer (Figure 5a). 

After installing the textile grid, 1800.0 mm-long plywood formwork was assembled over the textile, and the formwork was fixed to the slab by a set of L-shape angles and eight anchor bolts (Figure 5c,d). The anchor bolts (diameter = 5 mm, stainless steel) were installed in the slab at a spacing of 420–600 mm and 320 mm in the longitudinal and transverse direction of the specimens, respectively. As shown in Figure 5c, both sides of the formwork for the S-2 series specimen were directly fixed to the RC slab. On the other hand, two 20.0 mm-thick wood spacers were placed between both sides of the formwork and the slab for the S-1 series specimens to obtain a grout filling space (Figure 5e). 

The RC slabs were maintained in a water saturated state for 2 h and then the grout was filled manually, as shown in Figure 5f. The TRC strengthened specimens (SG series) were cured with a plastic covering in a temperature-controlled room at about 20 °C for 24 h and then steam-cured in a steam curing machine under atmospheric pressure for 8 h. Finally, the specimens were air-cured in a temperature-controlled room, at about 20 °C for 27 days. 

### 2.3. Test Setup

**Figure 7:**
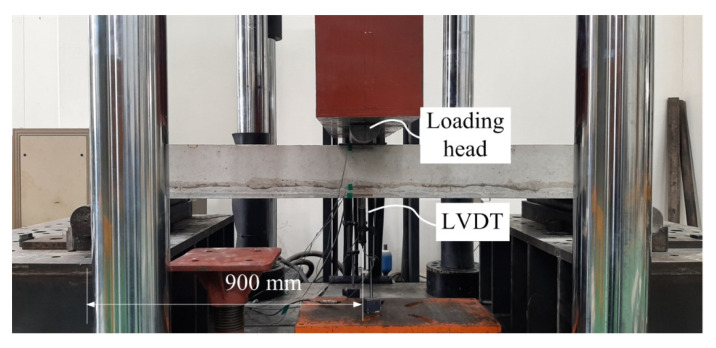
Flexural test setup and instrumentation.

As shown in Figure 7, a three-point bending test was conducted using a universal testing machine (capacity = 2000 kN) to examine the structural performance of the RC slabs strengthened with the TRC system. Static loading was applied to the specimen with displacement control at a speed of 1 mm/min. Two linear variable displacement transducers (LVDTs) were positioned at the mid-span of the specimen to measure the vertical displacement.

## 3. Test Results and Discussion 

### 3.1. Load-Displacement Behavior 

The results of a failure test for all sets of specimens are summarized in Table 5. As expected, concrete cracking load, steel yield load, and ultimate load of the slab specimens strengthened with the TRC system are increased to at least 170%, 113% and 124%, respectively, compared to the unstrengthened specimen. Note that the steel yield load was considered when the tensile strain went over 0.002 (= tensile strength 400 MPa/elastic modulus 200 GPa) simply that measured by a foil-type strain gauge mounted on the bottom bar. 

The SG-1 series specimens showed larger yield and failure loads than the SG-2 series specimens because the SG-1 series specimens are 20 mm thicker than the SG-2 series specimens.

**Figure 8:**
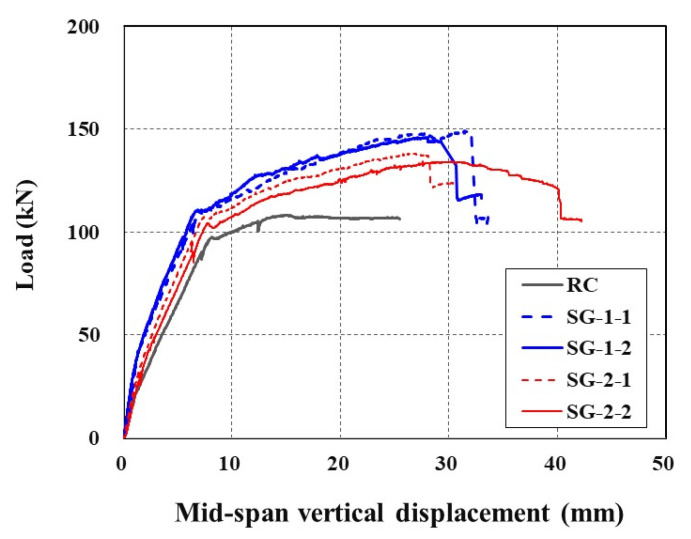
Load-displacement curves of specimens.

Figure 8 shows the applied load versus mid-span vertical displacements of all sets of specimens. The stiffness of the SG-1 and SG-2 series specimens until yield of steel reinforcement is at least 9% and 7% greater than that of the RC specimen. Under the same load level, the SG series specimens induce smaller displacement than the RC specimen does. The RC specimen exhibits shorter plastic deformation after steel yield than the strengthened specimens. Furthermore, the strengthened specimens were able to carry a significant level of additional load even after yield of steel reinforcement. This additional load carrying capacity beyond the yield of steel reinforcement is due to the TRC system. However, after the ultimate stage of loading, an abrupt failure occurred for the strengthened specimens due to rupture of the textile grid or debonding of the TRC system from the concrete substrate. The rupture of the textile grid at the ultimate loading stage was identified by a tearing sound of textile filaments during the test.

### 3.2. Crack Patterns and Failure Modes

**Figure 9:**
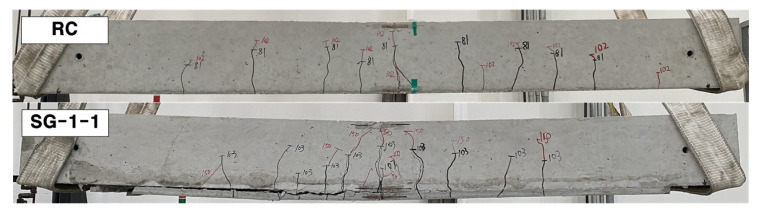

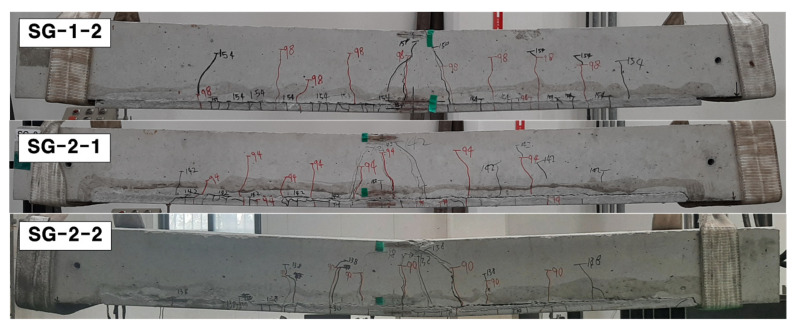
Crack patterns of specimens (side view).

Crack maps (side view) of the failure-tested specimens are provided in Figure 9. The RC specimen experienced pure flexural cracks and failed in flexural tension failure initiated by yielding of steel bars. It should be noted that the RC slabs are designed to be under-reinforced to maximize the strengthening effect. The RC slabs strengthened with the TRC system (SG series specimens) also experienced flexural cracks and failed either by flexure failure mode (SG-1-1, SG-1-2, and SG-2-1) or flexure failure mode followed by crushing of the top concrete (SG-2-2). All the SG series specimens finally showed debonding of the TRC system from the concrete substrate at failure. However, the debonding of the TRC system occurred after rupture of the textile. Therefore, the TRC system installed in this study appears to have sufficient bonding strength until failure. 

**Figure 10:**
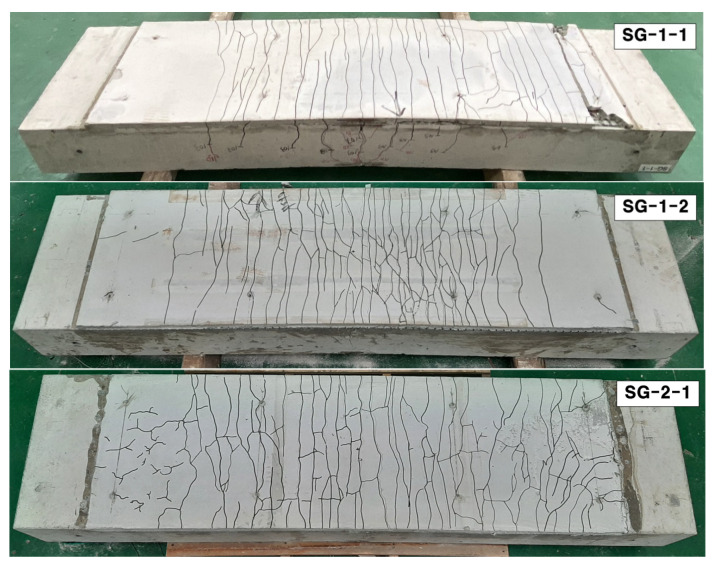

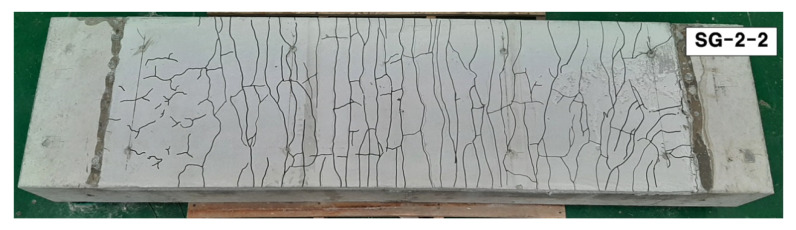
Crack pattern of specimens (bottom view).

Figure 10 presents crack maps (bottom view) of the specimens after completion of the failure test. The TRC system of all the specimens experienced numerous well-dispersed finer cracks than the RC slab. The strengthening of the RC slab with the TRC system was effective in terms of allowing the formation of multiple fine cracks within the TRC system while resisting the opening of major cracks in the RC slab.

### 3.3. Ultimate Load Carrying Capacity and Ductility 

The test results of this study are compared with those of a previous experimental study [17] to examine the influence of the strengthening method on the ultimate load carrying capacity and ductility of RC slabs strengthened with the TRC system. As provided in Table 6, the strengthening method employed for RC slabs in the previous study [17] involves the use of a precast TRC panel with grout. Except for the material properties of grouts, the textile reinforcement and sectional properties of the RC slab and the TRC system are the same.

**Figure 11:**
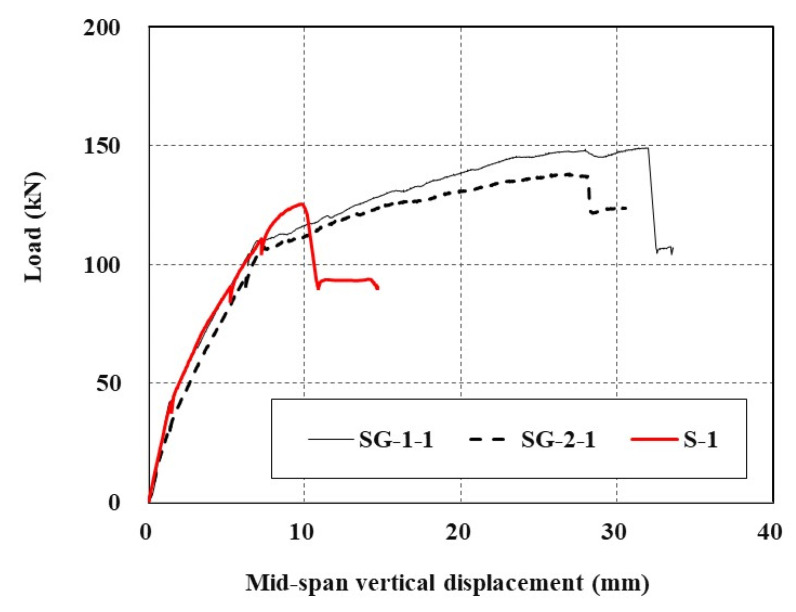
Comparison of load-displacement curves of TRC strengthened slabs.

**Figure 12:**
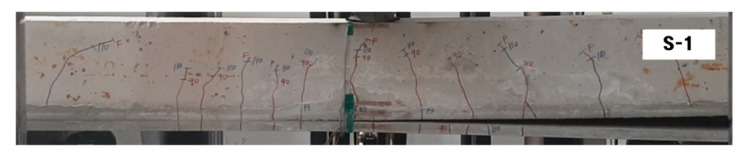
Failure mechanism of the S-1 specimen [17].

In Figure 11, the load versus mid-span vertical displacement curves for the specimens strengthened with the TRC system (SG-1 and SG-2 series) are compared with that of the precast TRC panel with grout [S-1]. Although the strengthening methods are different, the load-displacement behavior of the two sets of specimens is very similar until the load reached the yield of steel reinforcement. The SG-1 and SG-2 series specimens showed more ductile behavior beyond the yield of steel until failure than the S-1 specimen [17]. It should be noted that the S-1 specimen showed a sudden load drop after the load reached the ultimate failure. The abrupt failure of the S-1 specimen is due to the debonding of the TRC panel from the concrete substrate (Figure 12). 

**Figure 13:**
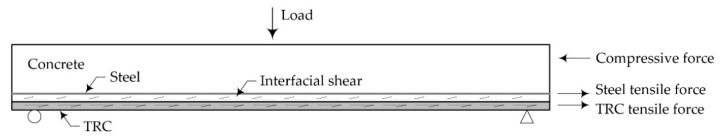
Load transfer mechanism of a RC flexural member strengthened with TRC system.

The debonding failure is one of the dominant failure modes of externally bonded strengthening methods including externally bonded Fiber Reinforced Polymer (FRP) systems [34] and TRC systems [35,36,37]. The debonding of the TRC system from the concrete substrate might be caused by the interfacial shear failure of the grout material (Figure 13) or by excessive curvature due to bending of the specimen at failure.

Debonding causes a brittle and catastrophic failure of strengthened structures. Therefore, various types of anchorage methods such as nailing, jacketing, anchor bolts, and spike anchors have been proposed in the literature to avoid debonding of FRP systems [38,39,40,41] and the TRC system [39] from the concrete substrate. As presented in Section 2.3, the plywood formwork was assembled to the RC slab by eight anchor bolts during the strengthening work and the anchor bolts remained after the strengthening work. Therefore, the TRC system was not only bonded to the RC slab by bonding strength but also was nailed to the slab by the anchor bolts. Furthermore, the grid anchors (Figure 5b and Figure 6) partially resist debonding stress of the TRC system from concrete substrate. 

It may be beneficial to examine the ductility of the RC slabs strengthened with the TRC system by a ductility factor. A displacement ductility factor (μ) can be defined as the ratio of displacement at ultimate load ($\Delta_{u}$) to displacement at yield load ($\Delta_{y}$), given as

(1) $$\mu =\frac{\Delta_{u}}{\Delta_{y}}$$

Table 7 summarizes the displacement ductility factors calculated for the RC slabs strengthened with the TRC system. The displacement ductility factors for the SG-1 and SG-2 series specimens are at least 3.3-times greater than that of the S-1 specimen [17]. Note that the precast TRC panel was directly bonded to the RC slab by grout for fabrication of the S-1 specimen. 

The greater ductility of the SG series specimens than the S-1 specimen can possibly be attributed to the anchor bolts. The TRC system of the SG series specimens was nailed to the RC slab by the anchor bolts. Therefore, the anchor bolts partially resist interfacial shear stress developed along the interface between the TRC system and the concrete substrate and provide additional load carrying capacity of the TRC system. The influence of the anchor bolts on the load carrying capacity and ductility is beyond the scope of this study. This should be investigated through an additional experiment program for specimens with and without the anchor bolts.

The SG series specimens have a simple load transferring mechanism, i.e., the RC slab–grout–textile reinforcement. On the other hand, the load transferring mechanism of the S-1 specimen is RC slab–grout-matrix (mortar)–textile reinforcement. Therefore, the SG series specimens have a simpler load transfer mechanism and fewer interfaces (structurally weak points) than the S-1 specimen.

## 4. Theoretical Analysis

In practice, to design a strengthening system or an RC flexural element with a TRC system, the structural behavior of a strengthened system should be analyzed by a theoretical method of analysis. In this study, the TRC strengthening effect was evaluated analytically in accordance with the ACI Design Guideline [4] and the obtained analytical solutions were compared with the test results. The objective of the theoretical analysis presented in this paper is to show that the behavior of a RC slab strengthened with a TRC system can accurately be estimated and thus the strengthening of RC flexural members with a TRC system can be designed through analytical calculation. The assumptions and methods including the constitutive models for concrete and steel and the equilibrium iteration to obtain the position of the neutral axis of a composite section employed in the theoretical analysis are the same as provided in Guideline [4] and as those presented in previous papers [26,42].

**Figure 14:**
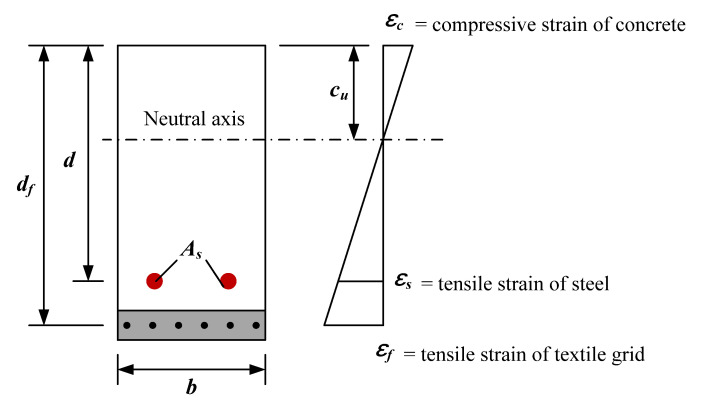
Internal strain distribution of a RC element strengthened by TRC system.

The ultimate moment ($M_{u}$) of the RC flexural element strengthened with the TRC system can be computed according to Equations (2)–(4) as follows

(2) $$M_{u}=M_{s}+M_{f}$$

(3) $$M_{s}=A_{s}f_{y}\left(d-\frac{\beta_{1}\left(c_{u}\right)c_{u}}{2}\right)$$

(4) $$M_{f}=A_{f}bE_{f}\varepsilon_{f}\left(d_{f}-\frac{\beta_{1}\left(c_{u}\right)c_{u}}{2}\right)$$

where $M_{s}$ and $M_{f}$ = ultimate moment by RC slab and TRC system, respectively; $A_{s}$ and $f_{y}\;$ = cross-sectional area and yield strength of longitudinal steel reinforcement, respectively; *d* and $d_{f}$ = distance from extreme compression fiber to centroid of tension steel and TRC system, respectively; $\beta_{1}$ parameter for computing the equivalent block stress; $c_{u}$ = distance from extreme compression fiber to neutral axis (Figure 14); $A_{f}$ = cross-sectional area of textile reinforcement (unit width); b = width of the cross section; and $E_{f}$ and $\varepsilon_{f}$ = modulus of elasticity and tensile strain of textile, respectively.

Steel yield load ($P_{y}$) and ultimate load carrying capacity ($P_{u}$) of the RC flexural element strengthened with the TRC system can be computed, respectively, by

(5) $$P_{y}=\frac{2M_{y}}{a}$$

(6) $$P_{u}=\frac{2M_{u}}{a}$$

where *a* = distance between load and support points. $M_{y}$ and $M_{u}$ = moment at steel yielding and ultimate that calculated by the tensile strength of steel and compressive strength of concrete, respectively.

In the theoretical analysis, two sets of material properties were employed. In the first set (A1), conservative material properties that are recommended in the design codes were assumed in the analysis, i.e., $f_{y}$ = 400 MPa [43] and ultimate tensile strain of textile ($\varepsilon_{fu}$) = 0.012 [4]. On the other hand, in the second set (A2), tested values of $f_{y}$ = 488 MPa and $\varepsilon_{fu}$ = 0.015 were assumed in the analysis. The compressive strength and ultimate compressive stain of concrete were assumed as 31 MPa and 0.003, respectively. 

In Table 8, the theoretical solutions are compared with the test data. When the material properties of A1 are used, the steel yield and ultimate loads are 11% and 5% underestimated on average relative to those of the average test results. On the other hand, when the material properties of A2 are used, the steel yield and ultimate loads are 8% and 13% overestimated than those of the average test results.

**Figure 15:**
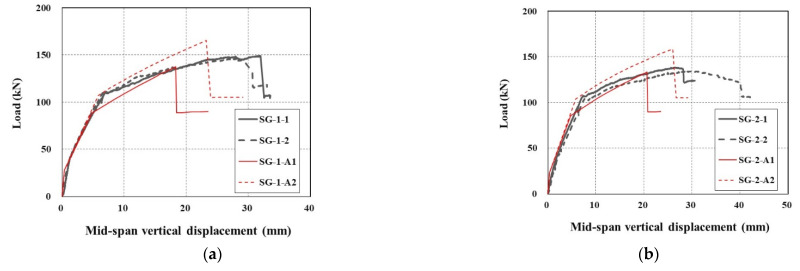
Comparison of theoretical solutions with test results: (**a**) SG-1 series; and (**b**) SG-2 series.

In Figure 15, the load versus mid-span vertical displacement curves of the specimens drawn by the theoretical solutions are compared with the test data. Overall, the steel yield load computed by a theoretical analysis showed a fair correlation with the test data. Furthermore, the ultimate load carrying capacity computed by a theoretical analysis with the material properties of A1 underestimates the test data but can be used for design purposes with a safety margin. 

## 5. Conclusions

This paper proposed an accelerated on-site installation method of a TRC system by grouting for repair and rehabilitation of deteriorated RC structures. The RC slabs were strengthened with a TRC system by the proposed installation method. Although removable formwork was required for the proposed method, the strengthening work was completed in a very fast manner by installation of textile grid and grouting. The proposed method could effectively be used to repair and rehabilitate RC structures with difficult accessibility or with a very narrow working space. 

To validate the effectiveness of the structural strengthening strategy and to examine the structural behavior, four RC slabs were strengthened with one ply of textile grid and 20 mm-thick grout. The TRC strengthened slab specimens were tested under flexure and the test results were compared with those of an unstrengthened specimen. The concrete cracking, steel yield and ultimate loads of the TRC strengthened slab are increased by at least 170%, 113% and 124%, respectively, compared to the unstrengthened specimen. Furthermore, the TRC strengthened specimens experienced longer plastic deformation after steel yield than the unstrengthened specimen. The TRC strengthened specimens exhibited many fine cracks and finally failed by rupture of the textile. Therefore, it can be concluded that the proposed installation method for the TRC system provides sufficient bonding strength between the TRC system and concrete substrate until failure. The improvement on bonding strength and ductility of tested specimens can partially be attributed to the anchor bolts and grid anchors.

The steel yield and ultimate loads of the TRC strengthened slabs were estimated by a theoretical analysis. The theoretically computed steel yield and ultimate loads overestimate the test data by 11% and 5%, respectively. Therefore, the response of the TRC strengthened slabs can be analyzed theoretically with good accuracy and the theoretical solutions can directly be used for design, if a proper safety margin is provided. 

Although the proposed method has been validated by a trial fabrication and structural failure test for RC slabs, several technical verifications should still be made for practical application. To evaluate the constructability of the proposed method for repair and rehabilitation of deteriorated RC structures, a demonstration project should be conducted and the total cost required for the proposed method should be compared with that of the conventional methods.

The proposed installation method of the TRC system uses removable formwork and anchor bolts that were mounted on the RC member and remained even after strengthening work. The anchor bolts partially resist interfacial shear stress developed along with the interface between the TRC system and the concrete substrate. To examine the influence of the anchor bolts on the load carrying capacity of TRC strengthened structural elements, an experiment program for specimens in flexure with and without the anchor bolts should be developed and conducted as future study. Moreover, a demonstration project needs to be conducted to validate the constructability of the proposed installation method of TRC system.

## Figures and Tables

**Table 1:** Mix composition of ready-mixed concrete (unit: kg/m^3^).

Cement	Water	Fly Ash	GGBS	Sand	Coarse Aggregate ^1^	Superplasticizer
263	167	56	56	828	934	2.63

^1^ Maximum grain size = 25 mm.

**Table 2:** Characteristic of slab specimens for flexural test.

Specimen ID	Indented Space	TRC Strengthening	No. of Specimens	Remarks
RC	No	No	1	Control
SG-1	No	Yes	2	-
SG-2	Yes	Yes	2	-

**Table 3:** Material properties of carbon textile grid (suggested by the manufacturer).

Fiber (tex) ^1^	Resin	Cross-Sectional Area of Textile ^2^ (mm^2^/m)	Tensile Strength (MPa)	Elastic Modulus (GPa)
3200	Epoxy	85	3300	220

^1^ tex = Grams per kilometer of yarn. ^2^ Cross-sectional area of yarn = 1.81 mm^2^.

**Table 4:** Mixture composition of grout (unit: kg/m ^3^).

Cement ^1^	Sand	Water	Silica Fume	Superplasticizer	Expansion Agent
1055.0	1130.0	142.0	42.0	8.4	99.8

^1^ Type I Portland cement specified in ASTM C150 [30].

**Table 5:** Results of failure test for RC slab specimens.

Specimen ID	Concrete Cracking		Steel Yielding		Ultimate Stage		Load Gain (%)	
	Load (kN)	Disp. (mm)	Load (kN)	Disp. (mm)	Load (kN)	Disp. (mm)	At Steel Yielding	At Ultimate Stage
RC	18.8	0.9	82.5	6.5	108.4	15.0	100	100
SG-1-1	42.4	1.1	103.2	6.4	149.0	31.2	125	138
SG-1-2	34.0	1.0	100.2	6.0	146.2	26.9	121	135
SG-2-1	32.0	1.4	93.9	6.4	138.3	26.3	114	128
SG-2-2	32.3	1.5	93.5	6.6	134.4	30.0	113	124

Note: Disp. = mid span vertical displacement.

**Table 6:** Characteristics of RC slabs strengthened with TRC system.

Specimen ID	Compressive Strength of Grout (MPa)	Dimensions of Specimen			Strengthening Method
		Width (mm)	Depth ^1^ (mm)	Ratio ^2^	
S-1 [17]	81.6	500	220	0.0075	Precast TRC panel with grout
SG-1 series	50	500	220	0.0075	CIP TRC system with grout
SG-2 series	50	500	200	0.0075	CIP TRC system with grout

^1^ Total depth including TRC system. ^2^ Steel reinforcing ratio (%).

**Table 7:** Displacement ductility factor for RC slabs strengthened with TRC system.

Specimen ID	Steel Yielding		Ultimate Stage		Ductility Factor
	Load (kN)	Displacement (mm)	Load (kN)	Displacement (mm)	
S-1 [17]	112.0	7.5	125.4	9.8	1.3
SG-1 series	101.7	6.2	147.6	29.1	4.7
SG-2 series	93.7	6.5	136.3	28.2	4.3

**Table 8:** Summary of theoretical solutions.

Material Property ID	Specimen ID	Theoretical Solutions				Theoretical Solutions/Test Data (%)			
		Steel Yield		Ultimate Stage		Steel Yield		Ultimate Stage	
		Load (kN)	Disp. (mm)	Load (kN)	Disp. (mm)	Load (kN)	Disp. (mm)	Load (kN)	Disp. (mm)
A1	SG-1	88.1	4.8	137.6	18.3	86.7	77.0	93.2	63.1
	SG-2	85.6	4.9	133.3	20.7	91.4	75.8	97.5	73.4
A2	SG-1	107.0	5.9	165.4	23.3	105.3	94.6	112.1	80.1
	SG-2	104.0	5.9	157.4	25.3	111.0	90.6	115.4	90.0

## Data Availability

The data presented in this study are available on request from the corresponding author.
